# Circulating tumor DNA dynamic variation predicts sotorasib efficacy in KRASp.G12C‐mutated advanced non‐small cell lung cancer

**DOI:** 10.1002/cncr.35917

**Published:** 2025-05-30

**Authors:** Francesco Passiglia, Francesco Pepe, Gianluca Russo, Edoardo Garbo, Angela Listì, Federica Benso, Claudia Scimone, Lucia Palumbo, Monica Pluchino, Roberta Minari, Paola Bordi, Massimiliano Cani, Antonio Ungaro, Chiara Ambrogio, Riccardo Taulli, Enrica Capelletto, Maurizio Balbi, Luisella Righi, Marcello Tiseo, Diana Giannarelli, Giancarlo Troncone, Silvia Novello, Umberto Malapelle

**Affiliations:** ^1^ Department of Oncology University of Turin S. Luigi Gonzaga Hospital‐Orbassano Turin Italy; ^2^ Department of Public Health University Federico II of Naples Naples Italy; ^3^ Department of Medicine and Surgery University of Parma Parma Italy; ^4^ Medical Oncology Unit University Hospital of Parma Parma Italy; ^5^ Medical Oncology Unit San Giuseppe Moscati Hospital Taranto Italy; ^6^ Department of Molecular Biotechnology and Health Sciences Molecular Biotechnology Center University of Turin Turin Italy; ^7^ IRCCS‐Facility of Epidemiology and Biostatistics Fondazione Policlinico Universitario A. Gemelli Rome Italy

**Keywords:** circulating tumor DNA, KRASp.G12C, liquid biopsy, non‐small cell lung cancer (NSCLC), sotorasib

## Abstract

**Background:**

The objective of this study was to investigate the correlation between circulating tumor DNA (ctDNA) *KRAS* G12C–mutation dynamic variations and treatment outcomes in patients with advanced non‐small cell lung cancer (NSCLC) receiving sotorasib therapy in a real‐world setting.

**Methods:**

Peripheral blood samples were prospectively collected from 32 patients at baseline, at cycle 3, and then at each radiologic assessment during sotorasib treatment. Both tissue and plasma samples were analyzed by using ultra‐deep, customized next‐generation sequencing (NGS) assays. Plasma samples from 27 of 32 patients also were analyzed by digital polymerase chain reaction analysis, and ctDNA dynamic variations were correlated with radiologic responses and patients' clinical outcomes.

**Results:**

A significant correlation between NGS and digital polymerase chain reaction–detected *KRAS* G12C variant allelic fractions (*p* < .001) was observed. Patients who achieved clearance of *KRAS* G12C–mutant ctDNA levels had a significant improvement in the objective response rate (80% vs. 8%; *p* < .001), median progression‐free survival (7.9 vs. 2.8 months; *p* < .001), and median overall survival (16.8 vs. 6.4 months; *p* < .001) compared with those who did not achieve clearance. The clearance of ctDNA was the only prognostic factor significantly associated with both median progression‐free survival (hazard ratio, 0.15; 95% confidence interval, 0.04–0.48) and median overall survival (hazard ratio, 0.09; 95% confidence interval, 0.02–0.45) in multivariable analysis. Moreover, a dynamic increase in the KRAS G12C median variant allele fraction anticipated radiologic disease progression in 70% of patients who were evaluable at the resistance time point.

**Conclusions:**

This study demonstrated that early clearance of *KRAS* G12C–mutant ctDNA predicted the clinical benefit of sotorasib in patients with advanced NSCLC, suggesting that dynamic monitoring of ctDNA levels also may anticipate sotorasib resistance.

## Abstract 2

Early clearance of circulating tumor DNA in patients who had advanced non‐small cell cancer with a KRASp.G12C mutation predicted benefit from sotorasib therapy. Conversely, a dynamic increase in the *KRAS* G12C–mutant variant allelic fraction anticipated disease progression on sotorasib in those with advanced disease.

## INTRODUCTION

Recently, an exon 2 p.G12C Kirsten rat sarcoma 2 viral oncogene homolog (*KRAS*) mutation was introduced in the arena of positive predictive biomarkers to be tested for treatment selection in patients with metastatic non‐small cell lung cancer (NSCLC).^1^ Sotorasib is the first‐in‐class covalent *off* inhibitor, able to irreversibly lock the KRAS G12C–mutated protein in its inactive state, impairing the downstream oncogenic signaling pathways.^2^, ^3^ Based on the positive results from the CodeBreak 200 and 300 trials (ClinicalTrials.gov identifiers NCT04303780 and NCT05198934, respectively),^4^, ^5^ sotorasib 960 mg daily was approved by the US Food and Drugs Administration in 2021 and by the European Medical Agency in 2022 for the clinical treatment of patients with *KRAS*p.G12C–mutant NSCLC who failed on previous immunotherapy and/or chemotherapy‐based regimens.^6^ Similar efficacy/safety outcomes were recently observed in the real‐world clinical setting in which different studies suggested the greatest benefit in patients with an Eastern Cooperative Oncology Group performance status (ECOG PS) of 0–1 and KEAP1 wild‐type disease and also in patients experiencing a partial response (PR) to sotorasib therapy.^7^, ^8^, ^9^ Liquid biopsy is a minimally invasive approach with multiple potential clinical applications in patients with lung cancer, including baseline tumor genotyping, acquired resistance tracking, as well as the longitudinal monitoring of disease burden during systemic treatments.^10^ Recent studies have demonstrated a high concordance rate between tissue and circulating tumor DNA (ctDNA)‐based genotyping as well as a good correlation between ctDNA dynamic variations and clinical outcomes in patients with advanced NSCLC undergoing both targeted therapies and immunotherapy.^11^, ^12^, ^13^, ^14^ Based on these data, a panel of experts recently proposed a plan toward the implementation of ctDNA response criteria in daily clinical practice as well as standardization of the Liquid Biopsy Response Evaluation Criteria in Solid Tumors (RECIST).^15^

In this rapidly evolving scenario, we sought to investigate the correlation between KRAS G12C dynamic variations and treatment outcomes in a cohort of patients with advanced NSCLC who were receiving targeted therapy with sotorasib in a real‐world clinical setting.

## MATERIALS AND METHODS

### Study design and treatment

This was a prospective, translational study conducted on patients with advanced, *KRAS*p.G12C–mutant NSCLC who were receiving sotorasib treatment in a real‐world clinical setting.

Patients were eligible if they were aged 18 years and older; had a histologically or cytologically confirmed diagnosis of NSCLC; stage IIIB–IIIC/IV disease (according to the eighth version of the American Joint Committee on Cancer/International Association for the Study of Lung Cancer tumor‐node‐metastasis [TNM] staging system); an ECOG PS < 3; a KRASp.G12C mutation; had disease progression or recurrence after receiving at least one prior systemic therapy for advanced/metastatic disease or were clinically unfit for first‐line standard regimens; received sotorasib 960 mg orally once daily until disease progression or unacceptable toxicity within the Italian Expanded Access Program from November 2020 to December 2022; participated in the PROMOLE translational study (part of a large scientific project called Digital Technology For Lung Cancer Treatment [DEFLeCT]), and signed and dated an informed consent and privacy form. Upon signing such a specific informed consent and privacy forms, patients indicated that they understood the purpose of and procedures required for the study and were willing to participate in the study, allowing the collection of both biologic samples and source data verification in accordance with Italian requirements, if applicable. Clinical, pathologic, and molecular data as well as treatment efficacy/tolerability outcomes were collected from the patients' medical charts and/or electronic health care records at the University of Turin and the University of Parma and were subsequently archived by using a specific electronic case report form. Radiologic evaluations of treatment efficacy using computed tomography scans were performed at baseline, at week 12, and every 12 weeks of therapy thereafter until disease progression and responses were evaluated by RECIST version 1.1.

The study was conducted in accordance with the International Conference on Harmonization Guidelines on Good Clinical Practice and the Declaration of Helsinki. The PROMOLE protocol was previously approved by the Independent Ethic Committee of S. Luigi Hospital, University of Turin (ethics approval number 73/2018 of 2024.01.30).

### Exploratory ctDNA analysis

Peripheral blood samples (ranging from two to eight collecting points) were taken from patients who were included in the PROMOLE translational study. Briefly, sampling collection was approached as follows: at baseline (day 1, cycle 1 of sotorasib administration) at cycle 3 (56 ± 10 days later), and at each radiological assessment thereafter (every 3 months) during treatment. Overall, two aliquots (2.0 ml) of plasma from each time point were isolated after two consecutive centrifugation steps (2300 revolutions per minute for 10 minutes) and shipped to the University of Naples for centralized molecular analysis. Moreover, aliquots from the same time point were batched in two distinct groups, arranging ctDNA purification as independent samples (batches 1 and 2, respectively). Each blood sample was processed for plasma collection according to standardized managing procedures and stored at −80°C until molecular analysis. Cell‐free DNA (cfDNA) isolation was performed using the QIAsymphony DSP Virus/Pathogen Kit (Qiagen) on the QIAsymphony SP/AS platform (Qiagen) starting from 1.2 mL of plasma, as previously validated.^16^ In detail, cfDNA was then eluted in a final volume of 60 μl nuclease‐free water and stored at −20°C until molecular analysis. Both tissue and plasma samples were analyzed by using a targeted next‐generation sequencing (NGS) approach. Specifically, tissue samples were tested by using the Ion Torrent platform (ThermoFisher Scientific) with the broad gene panel OCAv3 (Oncomine Comprehensive Assay, version 3; ThermoFisher Scientific), a solid‐tumor DNA and RNA kit assay covering 161 cancer‐associated genes in hotspot regions and full length, including copy number variation analysis and fusion detection. Liquid biopsy specimens were screened with an ultra‐deep, customized NGS assay capable of simultaneously analyzing 568 clinically relevant hotspot mutations in seven predictive biomarkers across different solid tumors.^16^ Overall, the exon 2 p.G12C *KRAS* hotspot mutation was analyzed with the SiRe panel for each clinically available ctDNA time points. Briefly, 20 μl of purified cfDNA was loaded on the Genexus platform (ThermoFisher Scientific), a fully automatized NGS system enabling entire NGS workflows (from library preparation to data interpretation) within 24 hours. Briefly, the SiRe panel covers hotspot mutations in seven clinically relevant predictive biomarkers (*EGFR*, *KRAS, NRAS*, *c‐KIT*, *BRAF*, *PIK3CA*, and *PDGFRA*) that play a pivotal role in the clinical stratification of patients with cancer. Concerning technical procedures, a sample list was created on a dedicated server and assigned to a new run. Customized primer pools, strip solutions, strip reagents, and supplies were loaded into the Genexus platform according to the manufacturer's instructions. For each sample, 20 µl of cfDNA was immediately dispensed on 96‐well plate following the manufacturer's instructions. Finally, nucleic acids were sequenced on GX5TM chip (ThermoFisher Scientific) that allowed the simultaneous processing of 16 samples in a single line with the customized SiRe assay. Data analysis was performed on proprietary Genexus software. Specifically, a dedicated bioinformatic pipeline built on technical requirements of the SiRe panel (assay size, genomic coordinates, alignment referenced and sequenced) was loaded using Ion Torrent Genexus analysis software (ThermoFisher Scientific). Coverage analysis was performed by adopting customized bed files on a proprietary Genexus plug in (coverage analysis, 5.16.0.4); variant annotations were automatically approached in accordance with the Ion Torrent Genexus analysis software (ThermoFisher Scientific). Of note, variants with 500X allele coverage and a quality score X20, within an amplicon coverage of at least 2000X alleles, were recorded. In addition, BAM files were also visually inspected with GenomeBrowse version 2.0.7 (Golden Helix) on lung cancer *EGFR*‐druggable, *KRAS*‐druggable, and *BRAF*‐druggable mutations. Of note, only mutations harboring a variant allele fraction (VAF) ≥0.2% were recorded for each examined time point; and, in case of discordant results between batches 1 and 2, the mean value was calculated as a reference value.^16^ Baseline detectable (VAF ≥0.2%), KRAS G12C–mutant ctDNA levels that became undetectable (VAF <0.2%) at the second time point were defined as *clearance*, whereas baseline KRAS G12C–mutant ctDNA levels that remained detectable were defined as *nonclearance*. Patients who were evaluable for ctDNA clearance analysis were required to have detectable KRAS G12C–mutant ctDNA at baseline and at least another plasma sample eligible for NGS analysis. Patients with undetectable KRAS G12C–mutant ctDNA at baseline were excluded from the ctDNA clearance analysis. Patients who were evaluable for an acquired resistance analysis were required to have liquid biopsy samples available at baseline and the time (within 30 days before) of progression on sotorasib. The overall spectrum of hotspot molecular alterations included in the SiRe panel was analyzed, and mechanisms of acquired resistance were defined as mutations that were absent at baseline and present at disease progression. A specific filtering algorithm was adopted, as detailed in Figure S1.

All available samples were also tested for exon 2 p.G12C *KRAS* hotspot mutations using the QIAcuity digital PCR (dPCR) system (QIAGEN) in accordance with the manufacturer's instructions. Briefly, 8 μl of cfDNA was manually loaded into the QIAcuity 26K 24‐well nanoplate (QIAGEN) to assess p.G12C *KRAS* hotspot mutations in a longitudinal series of plasma samples. Moreover, technical parameters (partitioning, processing, and fluorescent signal inspection) were automatically measured on proprietary software (QIAcuity software suite, version 1.2; QIAGEN). Finally, positive partitions for p.G12C mutation were calculated as follows: the number of DNA targets per partition (calculated from the Poisson distribution)/the number of valid partitions in each well.^17^

### Statistical analysis

The numbers and percentages of participants undergoing sotorasib therapy as well as their clinical, pathologic, and molecular characteristics were summarized either by descriptive statistics or as categorical tables. Descriptive analysis was performed, including means, standard deviations, medians, quartiles, and absolute/relative frequencies (with their respective two‐sided 95% confidence interval [CI] limits, where relevant), according to the specific variables. The Mann–Whitney test was used for intergroup comparisons of two independent samples, and the Fisher test was used for categorical values. Correlations were examined using the Pearson correlation coefficient. Radiologic evaluation of treatment efficacy was determined by computed tomography scanning every 12 weeks of therapy and thereafter until disease progression. The ORR was defined as the proportion of participants who had the best overall response of either a complete response (CR) or a PR as assessed by the investigators' review according to RECIST version 1.1. PFS was defined as the time from the date of treatment start until either disease progression, as assessed by the investigators' review according to RECIST version 1.1 criteria, or death from any cause, whichever occurred first. OS was defined as the time from the date of treatment start to death from any cause. The nonparametric Kaplan–Meier method was used to estimate survival curves. Medians and two‐sided 95% CIs were calculated, and Kaplan–Meier plots for both PFS and OS were obtained, as appropriate, with the use of the log‐rank test for comparisons, and a *p* value < .01 was set as threshold for statistical significance. In these analyses, patients were considered as censored observations if the event of interest (e.g., death or disease progression) did not occur as long as the patient was under observation, whereas patients were counted as failures if the event of interest occurred. Univariate and multivariate analyses were performed using the Cox proportional hazards and logistic regression models. The statistical analysis was performed using SPSS Statistics software, version 20 (IBM Corporation).

## RESULTS

### Patient characteristics

From November 2020 to December 2022, in total, 32 patients were included; their clinical characteristics are summarized in Table 1. The median patient age was 67.84 years (range, 46–81 years). The majority of the patients were men (53.13%), had an ECOG PS <2 (93.75%), and almost all were current or former smokers. Adenocarcinoma was the histologic subtype of the whole cohort. Tumor programmed death 1 (PD‐1) ligand (PD‐L1) expression was >50%, 1%–49%, and <1% in 18.75%, 50.0%, and 31.25% of patients, respectively. Most patients (78.13%) had stage IVB disease (extrathoracic metastasis). Bone was the most common metastatic site (34.38%) followed by the central nervous system (31.25%), and the liver (6.25%). Patients received a median of three lines of systemic therapies (range, from one to six lines), 34.38% received sotorasib in the first line, and 34.38% in the second line. In 59% of patients, the previous lines of treatment included immunotherapy with anti–PD‐1/PD‐L1 inhibitors. The median follow‐up calculated with the reverse Kaplan–Meier method was 32 months (range, 2–35 months) for the overall population at the time of data analysis.

**TABLE 1:** Baseline patient characteristics.

Patient characteristic	No. (%)
Age: Median [IQR/range], years	67.84 [61–74/46–81]
Older than 70 years	17 (53.12)
Younger than 70 years	15 (46.88)
Sex	
Men	17 (53.12)
Women	15 (46.88)
Smoking status	
Current	13 (40.63)
Former	18 (56.25)
Not available	1 (3.12)
ECOG performance status	
0	19 (59.38)
1	11 (34.38)
2	2 (6.25)
Histologic subtypes	
Adenocarcinoma	32 (100.00)
PD‐L1 expression level	
>50%	6 (18.75)
1%–49%	16 (50.00)
<1%	10 (31.25)
Metastatic sites	
Brain	10 (31.25)
Liver	2 (6.25)
Bone	11 (34.38)
No. of previous treatment lines for metastatic disease	
0	11 (34.38)
1	11 (34.38)
2	6 (18.75)
>3	4 (12.50)
Previous immunotherapy for metastatic disease	
Yes	19 (59.38)
No	13 (41.63)
Best response	
PR	10 (31.25)
SD	14 (43.75)
PD	8 (25.00)

Abbreviations: IQR, interquartile range; PD, progressive disease; PD‐L1, programmed‐death ligand 1; PR, partial response; SD, stable disease.

### Molecular findings by ctDNA analysis

**FIGURE 1:**
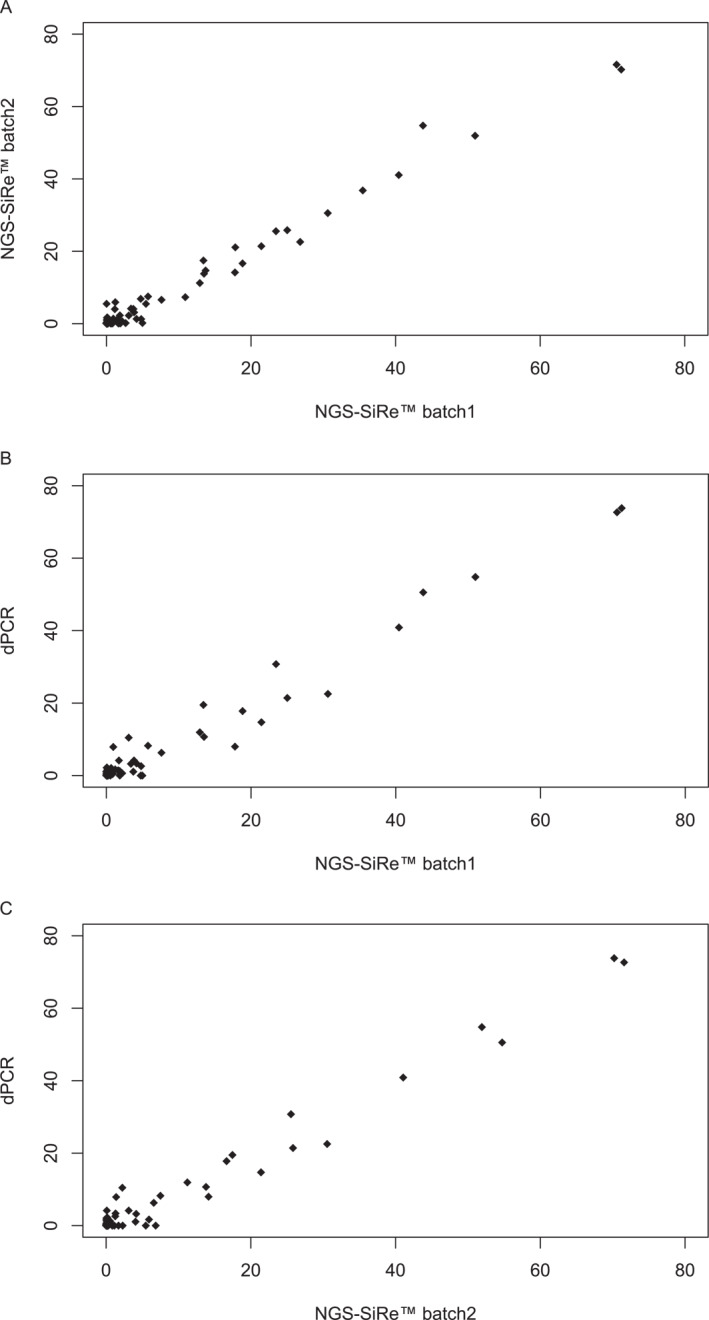
Overall correlation between (A) batch 1 and 2 NGS‐detected KRAS G12C VAFs, (B) batch 1 NGS and dPCR‐detected KRAS G12C VAFs, and (C) batch 2 NGS and dPCR‐detected KRAS G12C VAFs. dPCR indicates digital polymerase chain reaction analysis; NGS, next‐generation sequencing; VAFs, variant allele fractions.

All plasma samples were successfully analyzed by adopting an ultradeep NGS approach. Overall, for batching samples 1 and 2, we detected an average of 742,969.4 and 538,762.0 (Δ = 204,207.4) total reads (ranging from 374,818.0 to 1,234,962.0 and from 102,541.0 to 1,034,845.0 total reads, respectively); an average of 728,002.0 and 509,190.0 (Δ = 218,812.0) mapped reads (ranging from 357,157.0 to 1,217,950.0 and from 85,502.0 to 99,3451.0 mapped reads, respectively); a median read length of 123.7 and 113.9 (Δ = 9.8; ranging from 136.0 to 139.0 and from 94.0 to 128.0 median read length, respectively); an average reads per amplicon of 13,804.7 and 9199.0 (Δ = 4605.7; ranging from 5819.0 to 23,281.0 and from 1412.0 to 19,144.0 average reads per amplicon, respectively); a median percentage of 93.1% and 91.5% reads on target (Δ = 1.6; ranging from 76.1% to 98.0% and from 80.8% to 96.4% reads on target, respectively); a median coverage uniformity of 92.6% and 90.5% (Δ = 2.1; ranging from 87.9% to 94.0% and from 87.8% to 93.6% coverage uniformity, respectively). We observed a strong correlation between batch 1 and batch 2 NGS‐detected *KRAS*p.G12C VAFs (*r* = 0.99; *p* < .001; Figure 1A). Overall, exon 2 p.G12C *KRAS* hotspot mutations were detected in 11 of 32 (34.7%) baseline samples and in 11 of 24 (46%) resistance samples by inspecting automatic variant calling of analysis on Genexus integrated Variant Caller Interpretation software (version 5.16). Considering a technical cutoff of a 0.2% mutant allele fraction, visual inspection using the Golden Helix GenomeBrowse (version 2.0.7) revealed an exon 2 p.G12C *KRAS* hotspot mutation in 22 of 32 (69%) baseline NSCLC samples and in 20 of 24 (83%) resistance NSCLC samples.

Interestingly, p.G12C *KRAS* mutation was identified in 20 of 32 (62.5%) and 17 of 32 (53.1%) baseline samples from batch 1 and 2, respectively; whereas 16 of 24 (66.7%) and 19 of 24 (79.2%) resistance samples were p.G12C positive, respectively, integrating both automatized and visual inspection of molecular records. In addition, a median p.G12C VAF of 8.3% and 10.5% (Δ = −2.2; ranging from 0.2% to 51.0% and from 0.4% to 51.9%) was reported by inspecting baseline samples from batch 1 and 2, respectively; and a median p.G12C VAF of 20.8% and 17.2% (Δ = 3.6; ranging from 0.5% to 71.2% and from 0.2% to 71.6%) was detected in resistance samples from batch 1 and 2, respectively.

Plasma samples from 27 of 32 patients also were analyzed by dPCR. The median VAF detected by dPCR was 5.3% (ranging from 0.1% to 54.8%) for baseline samples and 10.4% (ranging from 0.2% to 72.6%) for the resistance samples. We observed a significant correlation between NGS and dPCR‐detected KRAS G12C VAFs (batch 1: *r* = 0.98; *p* < .0001 [Figure 1B]; batch 2: *r* = 0.99; *p* < .0001 [Figure 1C]).

### Sotorasib efficacy by baseline ctDNA KRASp.G12C mutation

All 32 patients included in the study were evaluable for tumor response assessment: 10 (31%) experienced a PR, 14 (44%) had stable disease (SD), and eight (26%) had progressive disease (PD) as their best response to sotorasib, with an ORR of 31% and a disease control rate (DCR) of 75%. At the time of data analysis (July 2024), four of 32 patients were still on treatment with sotorasib, and six of 32 patients were alive, with a median PFS of 5.8 months (95% CI, 4.1–7.5 months) and a median OS of 9.6 months (95% CI, 3.4–15.8 months) in the overall analyzed population. No significant differences in terms of the ORR (10% vs. 41%; p = .08), DCR (90% vs. 18%; *p* = .19), the median PFS (7.1 vs. 4.8 months; *p* = .29), or the median OS (9.6 vs. 13 months; *p* = .69) were observed between patients with undetectable (VAF < 0.2%) versus detectable (VAF > 0.2%) p.G12C KRAS mutations at the baseline ctDNA analysis (see Figure S2).

### Sotorasib efficacy by KRASp.G12C–mutant ctDNA clearance

**FIGURE 2:**
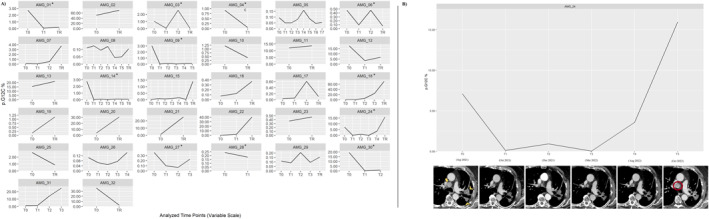
(A) Dynamic variations in KRAS G12C–mutant ctDNA (VAF) with the receipt of sotorasib therapy across 32 patients who had advanced NSCLC and were included in the study. An asterisk indicates patients who had ctDNA clearance. (B) A clinical case of paired molecular and radiologic evolution of disease during sotorasib therapy. The patient was affected by intrathoracic metastatic disease with detectable KRAS G12C–mutant ctDNA levels at baseline [T0]. After 1 month of sotorasib [T1], rapid clearance of KRAS G12C–mutant ctDNA was observed in correlation with a radiologic partial response of both mediastinal lymphadenopathy and left perihilar lesions (arrow and arrowheads, respectively). Subsequently, stable disease was noted with a relatively consistent level of KRAS G12C–mutant ctDNA. Finally, a rapid increase in the concentration of mutated ctDNA was registered before noticeable radiologic disease progression (mediastinal lymphadenopathy is indicated by the red circle). AMG, analysis mutated gene; ctDNA indicates circulating tumor DNA; T0‐6, Timepoints 0‐5; TR, timepoint resistance; VAF, variant allele fraction.

Among the 22 patients who had baseline detectable KRAS G12C–mutant ctDNA levels included in the study, 10 (45%) experienced a clearance of KRAS G12C–mutant ctDNA on an analysis of ctDNA levels at the second time point (Figure 2).

The ORR was 80% versus 8% (*p* < .001), and the DCR was 100% versus 42% (*p* = .003) in patients with and without clearance of KRASp.G12C–mutant ctDNA, respectively. In detail, eight of zero (89%), two of six (33%), and zero of seven (0%) patients who had ctDNA clearance experienced a PR, SD, and PD as their best response to sotorasib, respectively (*p* = .001).

**FIGURE 3:**
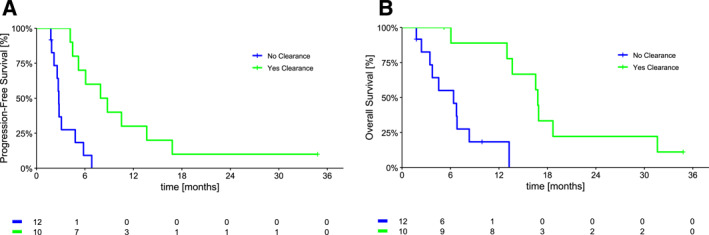
Median (A) progression‐free survival and (B) overall survival in patients who had clearance versus no clearance of KRAS G12C–mutant ctDNA under sotorasib therapy. ctDNA indicates circulating tumor DNA.

Patients who had clearance of KRASp.G12C–mutant ctDNA had a significant improvements in both median PFS (7.9 vs. 2.8 months; *p* < .001) and median OS (16.8 vs. 6.4 months; *p* = .001) compared with patients who did not have clearance (Figure 3).

The clearance of ctDNA was the only prognostic factor that remained significantly associated with both median PFS (HR, 0.15; 95% CI, 0.04–0.48) and median OS (HR, 0.09; 95% CI, 0.02–0.45) on multivariable analysis (Table 2).

**TABLE 2:** Univariate analysis of median overall survival and progression‐free survival.

Patient characteristic	HR (95% CI)	
	Overall survival	Progression‐free survival
Sex		
Men	1.53 (0.68–3.43)	1.12 (0.53–2.36)
Women	Ref	Ref
ECOG performance status		
0	Ref	Ref
1–2	1.94 (0.87–4.34)	2.02 (0.91–4.48)
Clinical stage		
IVA	Ref	Ref
IVB	1.01 (0.34–2.98)	0.84 (0.33–2.11)
PD‐L1 expression		
<1%	Ref	Ref
1%–49%	1.21 (0.49–2.97)	1.41 (0.61–3.22)
>50%	1.70 (0.58−4.98)	1.75 (0.63–4.89)
Brain metastasis		
No	Ref	Ref
Yes	0.83 (0.36–1.93)	0.93 (0.42–2.05)
Bone metastasis		
No	Ref	Ref
Yes	0.72 (0.31–1.68)	0.82 (0.37–1.81)
Previous immunotherapy		
No	Ref	Ref
Yes	1.29 (0.58–2.88)	0.91 (0.43–1.93)
No. of sotorasib lines		
1	Ref	Ref
2	0.95 (0.36–2.52)	0.94 (0.38–2.29)
≥3	1.39 (0.55–3.53)	0.99 (0.40–2.45)
KRASp.G12C detectability		
No	Ref	Ref
Yes	1.19 (0.51–2.74)	1.53 (0.69–3.38)
KRASp.G12C clearance		
No	Ref	Ref
Yes	0.09 (0.02–0.45)	0.15 (0.04–0.48)

Abbreviations: CI, confidence interval; ECOG, Eastern Cooperative Oncology Group; HR, hazard ratio; PD‐L1, programmed‐death ligand 1; Ref, reference category.

### Sotorasib resistance ctDNA analysis

Among the 28 patients who experienced PD according to RECIST during sotorasib therapy at the time of data analysis, 24 had a valid plasma sample collected within 3 months before radiologic evidence of disease progression and were then evaluable for the molecular resistance analysis. A dynamic increase of the KRAS G12C median VAF was detected in 16 of 24 patients (67%; five had undetectable mutations at baseline), with a median interval of 32 days (range, 24–40 days) between the detection of plasma molecular progression and radiologic evidence of disease progression (Figure 2).

In total, 110 mutations from 24 patients (median, 4.6 mutations; range, 1–13 mutations) were identified at the resistance collection time point after the filtering algorithm, as detailed in Figure S1. Specifically, 22 of 110 (20.0%) synonymous mutations were filtered out from the list because of low clinical impact. The remaining mutations (88 of 110 mutations; 80.0%) were distributed as follow: 53 of 88 (60.2%), 30 of 88 (34.1%), and four of 88 (4.5%) were *PIK3CA*, *NRAS*, and *ALK* nonsynonymous mutations, respectively; an *EGFR* mutation was identified in a single instance (one of 88 mutations; 1.2%). It is noteworthy that 77 of 88 (87.5%) missense mutations, 10 of 88 (11.4%) nonsense mutations, and one of 88 (1.2%) frameshift insertions were detected (see Table S1). In addition, a median mutant allele fraction of 28.1% (ranging from 1.2% to 96.5%) was observed. In total, 39 of 88 (44.3%%) unique molecular alterations (*n* = 21 *PIK3CA* [53.9%], *n* = 13 *NRAS* [33.3%], *n* = 4 *ALK* [10.2%], and *n* = 1 *EGFR* [2.6%] molecular alterations) were identified at the resistance time point in 13 of 24 (54.2%) patients with NSCLC.

### Sotorasib resistance tissue analysis

**FIGURE 4:**
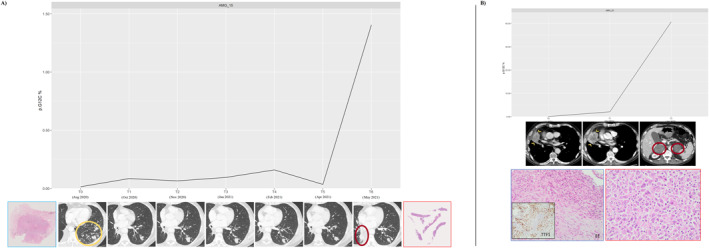
Paired molecular, radiologic, and morphologic evolution of disease during sotorasib therapy. (A) A patient who had an initial response to sotorasib in multiple left pulmonary nodules (yellow circle), followed by stable disease according to RECIST criteria, with a parallel low concentration of the mutant ctDNA at baseline (T0) and during the course of treatment. At the time of disease progression, a rapid increase in KRAS G12C–mutant ctDNA levels was recorded. Tissue‐core biopsy of the progressive right paravertebral lesion (red circle) confirmed the original adenocarcinoma histology of the resected specimen with KRAS G12C mutation. (B) A patient who had an initial, subtle, dimensional increase in the primary right pulmonary lesion (arrowheads) followed by RECIST disease progression characterized by the occurrence of new, bilateral, large adrenal masses (circles). At the ctDNA level, the progression corresponded to a rapid increase in mutant ctDNA levels. Histologic examination of the progressed ileal mass (see text) revealed a poorly differentiated sarcomatoid carcinoma rather than the original TTF1‐positive adenocarcinoma, resulting in a histologic shift of the tumor during sotorasib therapy. AMG, analysis mutated gene; ctDNA indicates circulating tumor DNA; EE, hematoxylin‐eosin; RECIST, Response Evaluation Criteria in Solid Tumors; T0‐6, timepoint 0‐6; TR, timepoint resistance.

A tumor tissue sample was available at the time of sotorasib resistance for two patients (Figure 4).

In the first patient (Figure 4A), the original diagnosis was made on a resected tissue sample consisting of a lobectomy with lymphadenectomy (August 2019) that revealed an adenocarcinoma subtype with a pT2N2 pathologic TNM stage and a KRASp.G12C mutation at molecular analyses. The patient then underwent adjuvant chemotherapy and radiotherapy from November 2019 to July 2020; however, they experienced early disease progression in the lung and brain, leading to the initiation of sotorasib in August 2020. Under sotorasib treatment, the best response to therapy was a partial response (multiple left pulmonary nodules indicated by a yellow circle in Figure 4A) followed by stable disease, with a parallel low concentration of the mutant ctDNA. Regrettably, in May 2021, PD featuring a right paravertebral mass was identified, and a CT‐guided core biopsy confirmed the adenocarcinoma histology and the presence of KRASp.G12C mutation.

In another patient (Figure 4B), a morphologic shift was observed under sotorasib therapy. In detail, at the time of the diagnosis, a TTF1‐positive lung adenocarcinoma was detected in a bronchial biopsy tissue, harboring KRASp.G12C mutations and PD‐L1 >50%. The patient received pembrolizumab in the first line and platinum‐pemetrexed in the second‐line and started third‐line therapy with sotorasib in June 2021. After 7 months of treatment with sotorasib (January 2022), the patient underwent an emergency ileal resection to prevent impending subocclusion. On gross examination, a mass extrinsically occluding the intestinal lumen was found, characterized by a malignant neoplastic proliferation with epithelioid and spindle cells with high atypia. Neither morphologic nor immunophenotypic features of adenocarcinoma were observed on microscopic examination. A pathologic diagnosis of undifferentiated cancer with sarcomatoid features was made. Molecular analyses confirmed the presence of the KRASp.G12C mutation, thus suggesting the metastatic nature of this lesion, likely originating from the primary lung adenocarcinoma. The patient definitively discontinued sotorasib because of a significant deterioration in clinical status (Figure 4B).

## DISCUSSION

This prospective study demonstrated that the early clearance of KRAS G12C–mutant ctDNA predicted increased tumor response as well as longer survival in patients with advanced NSCLC who received sotorasib treatment in a real‐world setting. Moreover, a dynamic increase in the KRAS G12C median VAF anticipated radiologic disease progression in 70% of patients who were evaluable at the resistance time point, suggesting a promising role of ctDNA dynamic monitoring for the clinical management of our patients.

The clinical interest in ctDNA as an early marker of tumor response/resistance is rapidly growing.^12^, ^13^, ^14^ Particularly in *KRAS* G12C–mutant disease, Paweletz et al. demonstrated that patients who had complete ctDNA clearance at cycle 2 had an improved ORR compared with those who had incomplete clearance.^18^ Conversely, Ernst et al. recently demonstrated that the DCR was significantly higher in molecular responders than in nonresponders, without any differences between complete versus incomplete KRAS G12C–mutant ctDNA clearance.^19^ In our study, 10 of 11 patients who experienced an objective response and 10 of 15 who attained disease control according to RECIST during sotorasib therapy had complete clearance of KRAS G12C–mutant ctDNA at cycle 3, suggesting that a complete molecular response could represent a more reliable marker than the relative change in the KRAS G12C VAF. Taking together, these data support the use of early ctDNA clearance to anticipate the likelihood of a favorable radiologic response and to guide intensification/de‐intensification strategies. Conversely, the standardized definition of molecular response remains a controversial point to be addressed in the near future as well as the optimal timing for ctDNA assessment during the disease course.^15^ In line with both Paweletz and colleagues^18^ and Ernst et al.,^19^ sensitivity for KRAS G12C ctDNA detection at baseline was approximately 70% in our study, which is in the range of ctDNA oncogene driver detection in patients with advanced NSCLC. Even if the ctDNA mutation VAF has been previously associated with tumor load and metastatic burden,^20^ pretreatment levels of detectable KRAS G12C–mutant ctDNA were associated with neither tumor response nor survival outcomes under sotorasib therapy. In this regard, the studies by both Paweletz et al.^18^ and Ernst and colleagues^19^ also indicated no difference in tumor response between pretreatment detectable versus undetectable KRAS G12C–mutant ctDNA. Conversely, Ernst et al.^19^ observed a significant correlation with patient survival, which suggests a prognostic rather than predictive role for baseline ctDNA detectability in this context. The results of our study also demonstrated that one third of patients who experienced a ctDNA increase in the of KRAS G12C median VAF before radiologic PD had undetectable mutations at baseline, suggesting that ctDNA monitoring could be considered for all the patients regardless of baseline ctDNA status. The technologies used for the detection of ctDNA are heterogeneous and affected by technical limitations.^21^ In this context, our data indicate that serial ctDNA monitoring with the targeted SiRe NGS panel is a feasible and reliable approach for the routine follow‐up of KRAS G12C mutation and also has similar accuracy compared with the dPCR platform. Finally, our study identified multiple potential resistance mechanisms across *PIK3CA*, *NRAS*, and *ALK*, and the pathogenetic role of these genes remains uncertain according to reporting from the current international knowledge base ClinVar. Unfortunately, we were not able to explore other resistance mechanisms described in recent studies^22^, ^23^ or the prognostic impact of co‐mutations across STK11/KEAP1 genes^24^ because they were not included in the SiRe NGS panel. We note that, to our knowledge, this is one of the first reports describing morphologic switching from adenocarcinoma to sarcomatoid cancer with an epithelial‐to mesenchymal transition (EMT) mechanism in a patients with KRAS G12C–mutant NSCLC who was undergoing sotorasib therapy.

Even if the low number of patients included in our study may represent a relevant limitation, the clinical characteristics, ORR, and survival outcomes of this population were similar to those reported in the CodeBreak 200 study^5^ and in other real‐world clinical series.^7^, ^8^, ^9^ Moreover, the confirmation of a statistically significant association between ctDNA clearance and patient outcomes in a limited study population, in our opinion, further support the reliability of this biomarker for clinical use, even if a more standardized approach is needed for a safe and effective implementation of liquid‐biopsy molecular response assessment in the real world.^15^

## AUTHOR CONTRIBUTIONS

**Francesco Passiglia**: Conceptualization; investigation; writing—original draft; methodology; validation; data curation. **Francesco Pepe**: Conceptualization; investigation; writing—original draft; methodology; validation; software; data curation; resources. **Gianluca Russo**: investigation; methodology; validation; resources; data curation. **Edoardo Garbo**: Investigation; methodology; validation; data curation. **Angela Listí**: Investigation; methodology; validation; data curation. **Federica Benso**: Validation; visualization. **Claudia Scimone**: Validation; visualization. **Lucia Palumbo**: Validation; visualization. **Monica Pluchino**: Validation; investigation; data curation. **Roberta Minari**: Investigation; validation; data curation. **Paola Bordi**: Investigation; validation; data curation. **Massimiliano Cani**: Validation; visualization. **Antonio Ungaro**: Validation; visualization. **Chiara Ambrogio**: Validation; visualization. **Riccardo Taulli**: Validation; visualization. **Enrica Capelletto**: Validation; visualization. **Maurizio Balbi**: Investigation; validation; data curation; writing—original draft. **Luisella Righi**: Investigation; validation; data curation; writing—original draft. **Marcello Tiseo**: Validation; visualization; supervision. **Diana Giannarelli**: Writing—original draft; validation; methodology; formal analysis; software; data curation. **Giancarlo Troncone**: Validation; visualization. **Silvia Novello**: Validation; visualization; supervision; funding acquisition. **Umberto Malapelle**: Conceptualization; investigation; funding acquisition; writing—original draft; validation; methodology; supervision.

## CONFLICT OF INTEREST STATEMENT

Francesco Passiglia reports personal/consulting and/or advisory fees from Amgen, AstraZeneca, BeiGene, Bristol Myers Squibb, Janssen, Merck Sharp and Dohme, Novartis, Pfizer, PharmaMar, Roche, and ThermoFisher Scientific outside the submitted work. Francesco Pepe reports personal/consulting and/or speakers' bureau fees from Menarini International and Roche outside the submitted work. Massimiliano Cani reports travel support from Amgen and AstraZeneca outside the submitted work. Chiara Ambrogio reports grants/contracts from Boehringer Ingelheim and Verastem Inc. outside the submitted work. Luisella Righi reports consulting/advisory fees from AstraZeneca, Boehringer Ingelheim, Eli Lilly & Company, Novartis, and Roche outside the submitted work. Marcello Tiseo reports institutional research grants from AstraZeneca, Boehringer Ingelheim, and Roche; personal/consulting and/or speakers' fees from Amgen, AstraZeneca, BeiGene USA Inc., Boehringer Ingelheim, Bristol Myers Squibb, Daiichi Sankyo, Eli Lilly & Company, F. Hoffmann‐La Riche, Johnson & Johnson Health Care Systems Inc., Merck, Merck Sharp and Dohme, Novartis, Pfizer, Pierre Fabre Pharmaceuticals, and Takeda Oncology; and travel support from Amgen and Takeda Oncology outside the submitted work. Diana Giannarelli reports personal/consulting and/or advisory fees from AstraZeneca, Amgen, and Sanofi outside the submitted work. and Amgen. Giancarlo Troncone reports personal advisory or speakers' fees from Bayer, Merck Sharp & Dohme, Roche, and Pfizer outside the submitted work. Silvia Novello reports grants/contracts from Amgen, AstraZeneca, BeiGene Switzerland GmbH, Boehringer Ingelheim, Eli Lilly and Company, F. Hoffman La‐Roche, Merck Sharp and Dohme, Novartis, Pfizer Canada Inc., and Takeda Oncology; and support for professional activities from AstraZeneca, BeiGene Switzerland GmbH, Bristol Myers Squibb, Novartis, Takeda Oncology, and ThermoFisher Scientific outside the submitted work. Umberto Malapelle reports personal/consulting or speakers' bureau fees from AstraZeneca, Amgen, Boehringer Ingelheim, Diaceutics, Diatech, Eli Lilly & Company, GlaxoSmithKline, Hedra, Janssen Biotech, Merck, Merck Sharp & Dohme, Novartis, Roche Health Solutions Inc., and ThermoFisher Scientific outside the submitted work. The remaining authors disclosed no conflicts of interest.

## Supporting information

Supplementary Material

Supplementary Material

Supplementary Material

Supplementary Material

Supplementary Material

## Data Availability

Additional data are provided in the Supporting Information section of this article.
