# Supplementary material for: Circulating tumor DNA dynamic variation predicts sotorasib efficacy in KRASp.G12C‐mutated advanced non‐small cell lung cancer
Source: Cancer. 2025 May 30;131(11):e35917. doi: 10.1002/cncr.35917 (PMC12124469; doi:10.1002/cncr.35917)
Supplement: Supplementary file 1 — Supplementary Material [file CNCR-131-e35917-s005.docx]

**FIGURE S1.** Filtering methodological workflow used for the analysis of molecular alterations identified by circulating tumor DNA analysis at time of disease progressive to sotorasib.

**FIGURE S2.** Median progression-free survival (A) and median overall survival (B) in patients with a detectable (VAF>0.2%) versus undetectable (VAF<0.2%) baseline ctDNA KRASp.G12C mutation.
