# Supplementary material for: Circulating tumor DNA dynamic variation predicts sotorasib efficacy in KRASp.G12C‐mutated advanced non‐small cell lung cancer
Source: Cancer. 2025 May 30;131(11):e35917. doi: 10.1002/cncr.35917 (PMC12124469; doi:10.1002/cncr.35917)
Supplement: Supplementary file 2 — Supplementary Material [file CNCR-131-e35917-s001.docx]

**TABLE S1.** Molecular alterations identified by ctDNA analysis at the time of disease progression to sotorasib across 32 advanced NSCLC patients included in the study
