# Supplementary figures and images for: Circulating tumor DNA dynamic variation predicts sotorasib efficacy in KRASp.G12C‐mutated advanced non‐small cell lung cancer
Source: Cancer. 2025 May 30;131(11):e35917. doi: 10.1002/cncr.35917 (PMC12124469; doi:10.1002/cncr.35917)

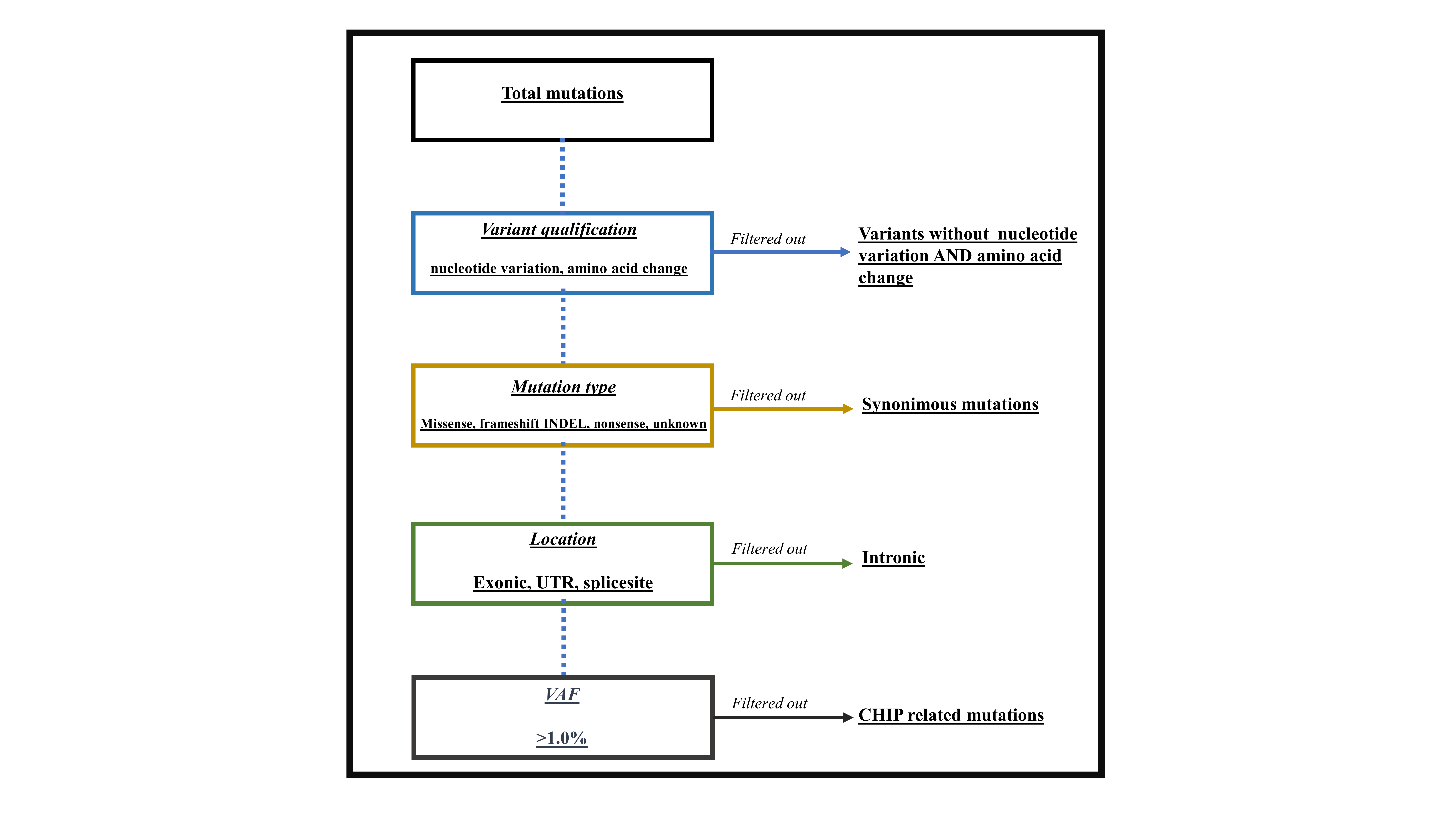

Supplement: Supplementary file 4 — Supplementary Material [file CNCR-131-e35917-s002.TIF]

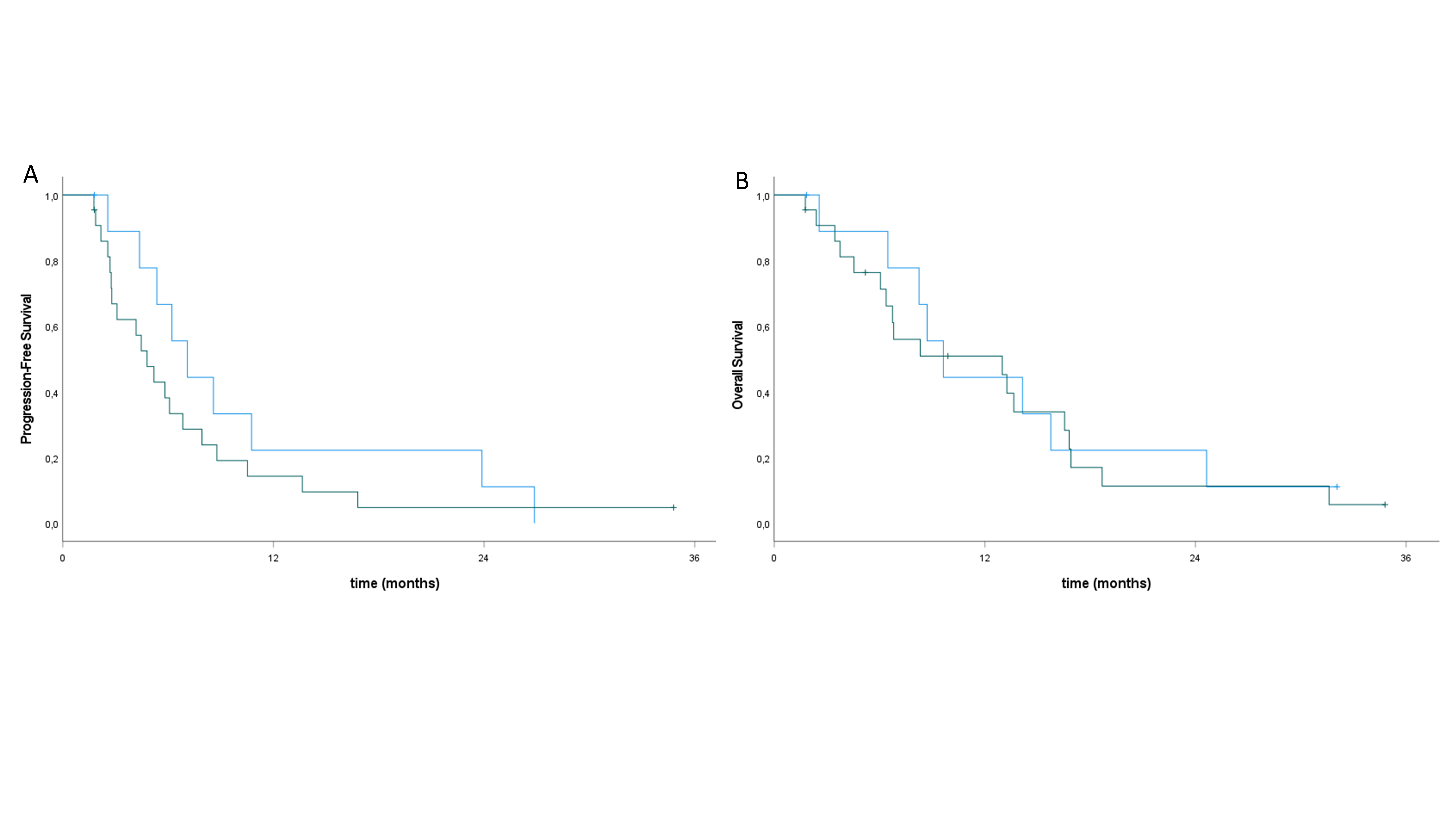

Supplement: Supplementary file 5 — Supplementary Material [file CNCR-131-e35917-s004.TIF]
